# Correlations between childhood maltreatment, anxiety and depressive symptoms, and risk behaviors in adolescent schoolchildren

**DOI:** 10.47626/2237-6089-2021-0456

**Published:** 2024-02-06

**Authors:** Daniela Ladeira Reis, Mônica Gonçalves Ribeiro, Isabela Couto, Nina Maia, Dagoberto Bonavides, Ana Cristina Botelho, Claudia Luisa Sena, Curt Hemanny, Irismar Reis de Oliveira

**Affiliations:** 1 Programa de Pós-Graduação em Processos Interativos dos Órgãos e Sistemas Instituto de Ciências da Saúde Universidade Federal da Bahia Salvador BA Brazil Programa de Pós-Graduação em Processos Interativos dos Órgãos e Sistemas, Instituto de Ciências da Saúde, Universidade Federal da Bahia (UFBA), Salvador, BA, Brazil.; 2 Instituto Psicoeducar Terapias Cognitivas Salvador BA Brazil Instituto Psicoeducar Terapias Cognitivas, Salvador, BA, Brazil; 3 Faculdades Integradas Padrão Guanambi Guanambi BA Brazil Faculdades Integradas Padrão Guanambi (FIPGuanambi), Guanambi, BA, Brazil.; 4 Departamento de Neurociências e Saúde Mental Faculdade de Medicina UFBA Salvador BA Brazil Departamento de Neurociências e Saúde Mental, Faculdade de Medicina, UFBA, Salvador, BA, Brazil.

**Keywords:** Maltreatment, depression, anxiety, mental health, adolescence

## Abstract

**Objective:**

Childhood maltreatment is extremely harmful to health, especially in relation to development of the psychiatric disorders throughout life. The objective of this study was to describe the prevalence and types of maltreatment in a sample of adolescent schoolchildren and to investigate associations between maltreatment types and anxiety and depressive symptoms, sociodemographic variables, and risk behaviors. The study also identified which variables were the greatest predictors of anxiety and depressive symptoms.

**Methods:**

We conducted a cross-sectional study with a sample of 654 school students aged 11 to 17 years. We collected sociodemographic data and administered the Revised Child Anxiety and Depression Scale (RCADS-47) to measure anxiety and depressive symptoms as well as the Childhood Trauma Questionnaire (CTQ) to evaluate maltreatment and adverse experiences such as abuse and negligence during childhood and adolescence. Statistical analyses were conducted to estimate correlations between sociodemographic data, anxiety, depression, and types of maltreatment. A regression analysis was also conducted to identify maltreatment types that predict psychological symptoms.

**Results:**

Emotional abuse and emotional neglect were the most prevalent types of maltreatment. Statistically, emotional abuse was the maltreatment type most strongly correlated with depression and anxiety and tended to co-occur with other types of maltreatment. Additionally, emotional and sexual abuse were the greatest predictors of anxiety and depression in adolescents.

**Conclusion:**

The above results reinforce the findings of previous studies in terms of understanding the effects of maltreatment. They identify emotional abuse as the main predictor of depressive and anxiety symptoms.

## Introduction

Childhood maltreatment encompasses actions, omissions, and threats, usually made by those directly responsible for the child, causing them physical, psychological, and sexual damage, and impairing their development.^1^ The most common types of maltreatment found in the literature are emotional abuse, physical abuse, sexual abuse, emotional neglect, and physical neglect. Interpersonal maltreatment experiences involve offensive, critical, and invalidating attitudes, communicating unlove and non-belonging, physical punishments, sexual coercion, and denial of the child’s affective and physiological needs.^2-5^

Children exposed to maltreatment are subject to neurobiological, social, emotional, cognitive, and behavioral changes, which make them vulnerable to the onset of physical diseases (such as cancer, autoimmune diseases, asthma, type 2 diabetes, metabolic changes, and cardiovascular diseases)^6^ and especially to mental disorders – of which, anxiety, depression, and borderline personality disorder are the most documented in the literature.^7,8^ This population frequently has emotional regulation difficulties and functional losses in interpersonal, family, and school relationships.^9,10^

Maltreatment occurring in childhood and youth produces effects throughout development, leading to anxiety and depression symptoms and, more specifically, impairing the formation of identity and personality. The effects can impair the role transition that occurs throughout development and adulthood, as people grow into socio-functional life.^10,11^

In 2015, approximately 1.7 billion children worldwide suffered interpersonal violence. In Brazil, 17,900 cases of violence against children were identified, most of them committed by their parents,^12^ although violence is also committed by other family members and at school, through virtual media, and on the street.^13^

Family and social bonds in the context to which adolescents belong make important contributions to development.^14^ Some authors^15^ suggests that construction of healthy relationships with the world is mediated by attachment figures. Therefore, responsive parents – i.e., healthy attachment figures – promote development of self-assurance and more adaptive relationships in developing youth. On the other hand, young people who have been mistreated by attachment figures are more vulnerable to mental health problems.^16^ There is a growing rate of psychiatric disorders in adolescence, with anxiety and depression being the most prevalent.^17^ Research also indicates that mental disorders arise from the interaction between biological and environmental factors.^18,19^ In this sense, data show several correlations between maltreatment in childhood and psychiatric disorders that develop in adolescence and adulthood.^20,21^

Studies indicate that anxiety disorders and depressive disorders (internalizing disorders), substance use disorders and antisocial behavior (externalizing disorders), and also suicidal behaviors are the most associated with and predicted by cases of abuse and neglect.^14-17^ Being exposed to these harmful acts at an early age generates greater symptom chronicity and duration.^18,19^

Symptoms that emerge due to abuse and neglect are heterogeneous. They vary in terms of symptom types, the form of clinical presentation, severity, and evolution. Hence, correlating maltreatment to various anxiety and depressive symptoms poses a challenge for research, while developing strategies for more effective treatment poses a challenge to clinical practice.^20^ The relationship between abuse and symptoms seems to be mediated by other risk or protection factors, such as genetic characteristics, life history, attachment, and cultural characteristics.^21-23^ Thus, the literature also describes cases of resilience to maltreatment, although at a smaller proportion than the negative impacts.

Maltreatment exposure is manifold, influencing symptom heterogeneity and constituting an encompassing field of research.^6^ This is because the data are still too inconsistent to explain which is more harmful: suffering only one specific type of maltreatment (e.g., emotional abuse or sexual abuse alone) or being subject to several simultaneous types of maltreatment (e.g., concomitant physical and sexual abuse), or whether their effects are dependent on the variety of types of maltreatment suffered.^6^ There is not yet consensus on whether the different types of maltreatment produce externalizing or internalizing disorders.^6,24^ For instance, physical abuse has been associated with development of externalizing symptoms and disorders, namely: conduct disorders, impulsivity, anger, aggressiveness, disruptive behavior, and criminal behavior.^25,26^ Other research indicates that emotional abuse more often produces mood disorders, such as major depressive disorder and bipolar disorder, whereas sexual abuse predicts occurrence of borderline personality disorder.^26^

A study conducted by Cecil et al.,^6^ incorporated all types of maltreatment as covariables and described their specific, cumulative, and shared effects on the occurrence of psychiatric symptoms. The sample comprised 204 adolescents and young adults at high risk of exposure to violence, poverty, and drugs in the United Kingdom. Firstly, the results indicated that all types of maltreatment were mutually correlated and that they were more commonly simultaneous. Secondly, the more abuse and neglect were suffered, the more severe the symptoms became. Thirdly, shared effects indicated that all types of maltreatment were associated with psychological symptoms, although emotional abuse was the harm most predictive of symptoms and was mediated by exposure to violence and victimization. The study suggested replications should be conducted with other populations to reinforce the findings.

In this regard, a study by our group using the same methodology^24^ analyzed 347 school-aged high-risk adolescents, exposed to violence, poverty, drugs, and drug trafficking. In agreement with the previous study, the results indicated that the different types of maltreatment frequently co-occur, that there is a direct relationship between the various types of maltreatment and the severity of the symptoms, and that emotional abuse was the main indicator of anxiety and depression symptoms. The authors discussed limitations associated with the specificity of emotional abuse and sample biases.

Based on the need for studies in a population vulnerable to frequent maltreatment, which is associated with anxiety and depressive symptoms, this study replicated part of the methodology of the two abovementioned studies and sought to I) identify the prevalence of the maltreatment types in a sample of low-risk school adolescents, II) describe the main correlations between different maltreatment types and psychological symptoms, and III) identify which types of maltreatment increase the probability of occurrence of anxiety and depressive symptoms.

## Methods

### Study design and participants

This cross-sectional study collected data on demographic variables, anxiety and depressive symptoms, and frequency and types of childhood maltreatment. The sample comprised students from a public school in Salvador, the fourth largest city in Brazil.

Participants were adolescents aged 11-17, who provided data on childhood maltreatment. Students in this study comprised grades 6 through 12 (middle and high school).

### Procedures

We replicated the methods used in two previous studies^6,24^ that investigated the presence of maltreatment and psychological symptoms in Brazilian and British adolescents. In the present study, we investigated a sample of students from a Brazilian public school. Here, we studied a sample of adolescent schoolchildren, although different from the samples in the previous two studies in that these adolescents were at lower risk of violence.^27^

We collected data during the first and second semesters of 2017. After approval by the school principal, the project was presented to parents and teachers. The parents read and signed an informed consent form. Subsequently, we introduced the project to the students, answering their questions, and inviting them to sign an assent form. Lastly, the students filled out the questionnaires and inventories in the classroom.

### Assessment instruments

#### Sociodemographic variables

A questionnaire was used that covers sex, color or race, and risk behaviors (self-cutting, drug use, and bullying).

#### Risk behaviors

The frequency of self-reported bullying victimization and perpetration over the past term was assessed with the two global items of the Olweus Bully/Victim Questionnaire (OBVQ)^24,28-30^: (“How often have you been bullied?” and “How often have you taken part in bullying other students?”). The items were coded as: 0 = never; 1 = once or twice; 2 = two or three times per month; 3 = about once per week; or 4 = several times per week. Students were also asked to report on their drinking of alcohol, use of cannabis, and use of other street drugs over the past 6 months, as well as self-cutting or self-harm behaviors. Risk behaviors were classified as categorical variables in the data analysis (no/never = 0; yes = 1).

#### Anxiety and depressive symptoms

The Revised Child Anxiety and Depression Scale (RCADS-47)^31,32^ was used to assess anxiety and depressive symptoms. This self-administered scale comprises 47 questions on the presence of anxiety and depressive symptoms, scored from 0 (never) to 3 (always). It is subdivided into six subscales: social phobia, separation anxiety, obsessive-compulsive disorder, panic disorder, generalized anxiety disorder (the sum of which defines the total anxiety score), and major depressive disorder. The crude total score (the sum of anxiety and depressive symptoms or internalizing symptoms) is converted into a T score, and values above 65 indicate threshold symptoms, while values over 70 indicate clinical symptoms (https://www.childfirst.ucla.edu/resources/).

#### Maltreatment

For the assessment of childhood maltreatment, we utilized the Childhood Trauma Questionnaire (CTQ),^30^ which has been translated into Portuguese and adapted for Brazilian populations.^33^ This instrument includes 28 questions with a response scale ranging from 1 to 5 points. The 28 questions are subdivided into five types of maltreatment: emotional abuse, physical abuse, sexual abuse, emotional neglect, and physical neglect. Each occurrence of maltreatment is classified according to its score and severity: none to minimal, low to moderate, moderate to severe, or severe to extreme.^22,30^

## Statistical analysis

We calculated clinical and demographic descriptive statistics for the sample (n, percentage, mean, and standard deviation). Additionally, we computed bivariate correlations between the types of maltreatment and the clinical (anxiety and depressive symptoms) and demographic variables (age, sex, and color/race).

We also provided descriptions of the prevalence, in absolute numbers and percentages, of each type of maltreatment reported by the sample (emotional abuse, physical abuse, sexual abuse, emotional neglect, and physical neglect). Furthermore, we evaluated the severity of each type of abuse and neglect as categorical variables (minimal, moderate, severe, or extreme) and determined the numbers of adolescents who suffered one or more types of abuse simultaneously.

To test correlations between categorical variables, like types of maltreatment and sociodemographic variables, we computed Spearman’s correlation coefficients (*ρ* or rho).

In terms of categorizing sex and color/race as dichotomous variables, we assigned a code of 0 to females and 1 to males, and a code of 1 to white individuals and 0 to non-white individuals. This categorization process was similarly applied to other racial and ethnic categories, including black, East Asian, indigenous, and multiracial, to facilitate frequency assessments and correlation calculations.

Using multiple linear regression, we investigated the interactions between all types of maltreatment (predictor variables) and each anxiety and depression symptom (criterion variables). Consistent with the methodology of replicated studies,^6,24^ these analyses first considered each type of maltreatment alone as a predictor. To enhance the identification power for the most strongly predictive types of maltreatment, we incorporated all maltreatment types as covariables in our analyses. We conducted binary logistic regression analyses with all types of maltreatment, age, sex, and ethnic group as covariables to assess the types of maltreatment as predictors of cannabis, alcohol, and other substance use, bullying others, and engaging in self-harm (all dichotomous variables). All regression analyses included sex, age, and ethnic groups as covariables. The statistical significance level was established at 0.05, with 95% confidence intervals. All the analyses were performed with SPSS, v. 24.^34^

## Ethical aspects

This study forms a component of a larger research project comparing the efficacy of group trial-based cognitive training (G-TBCT) in reducing anxiety and depressive symptoms in school adolescents. It secured approval from the Maternidade Climério de Oliveira Institutional Review Board, Universidade Federal da Bahia, (evaluation report no. 3.024.360).

## Results

### Sample characteristics

A total of 684 students signed the assent and consent forms; however, 30 of them (4.3%) did not attend for the procedures or refused to fill in the questionnaires. The sample comprised 654 students, with 346 (52.9%) identifying as female. Mean age was 14.3 years. The demographic and clinical sample characteristics are shown in Table 1.

**Table 1:** Clinical and sociodemographic sample characteristics

Variable	n (%)
Age: mean (SD)	14.34 (1.89)
Sex/female	346 (52.9)
Ethnicity	
Multiracial	290 (44.4)
Black	194 (29.7)
White	95 (14.5)
East Asian	43 (6.6)
Indigenous	30 (4.6)
Has considered self-cutting	
Never	468 (71.7)
Once or twice	122 (18.7)
Three times or more	62 (9.5)
Has intentionally self cut	
Never	549 (84.2)
Once	60 (9.2)
Twice or more	43 (6.6)
Alcohol use	
Never used	362 (55.4)
Used once or twice	234 (35.8)
Used 2 to 4 times in the last month	49 (7.5)
Used more than once a week	9 (1.4)
Cannabis use	
Never used	643 (98.5)
Used once or twice	10 (1.5)
Has been bullied	
Never	443 (67.7)
Once or twice	160 (24.5)
2 to 3 times in the last month	24 (3.7)
Once a week	6 (0.9)
Has bullied others	
Never	495 (75.7)
Once or twice	100 (15.3)
Two or three times	21 (3.2)
Once a week	17 (2.6)
Many times a week	21 (3.2)

SD = standard deviation.

### Main results: maltreatment prevalence

The prevalence of abuse and neglect suffered by students is shown in Table 2. It also shows the number of types of abuse suffered concomitantly. We found that emotional abuse was the most frequent type of severe maltreatment (8.9%). Less than half of the sample (35.9%) had not suffered any type of maltreatment.

**Table 2:** Prevalence of maltreatment in the sample

	Prevalence in the sample (%)*	Prevalence in the sample (%)^†^	CTQ score classification			

			None or minimal % (n)	Low to moderate % (n)	Moderate to severe % (n)	Severe to extreme % (n)
Types of maltreatment						
Emotional abuse	42.4	18.2	57.6 (376)	24.2 (158)	9.3 (61)	8.9 (58)
Physical abuse	14.8	6.9	85.2 (554)	7.8 (51)	4.3 (28)	2.6 (17)
Sexual abuse	10.2	3.3	89.8 (583)	6.9 (45)	2.8 (18)	0.5 (3)
Emotional neglect	39.0	11.6	61.0 (399)	27.4 (179)	7.0 (46)	4.6 (30)
Physical neglect	14.5	4.5	85.5 (553)	10.0 (65)	4.0 (26)	0.5 (3)
					
Number of simultaneous maltreatment types experienced		Number of adolescents % (n)				
0		35.9 (225)				
1		26.0 (170)				
2		22.3 (146)				
3		10.9 (71)				
4		4.6 (30)				
5		0.3 (2)				

CTQ = Childhood Trauma Questionnaire.

* Including low, moderate, and severe cases of maltreatment.

^†^ Including only moderate and severe cases.

### Secondary results: correlations between maltreatment and clinical and sociodemographic characteristics

Bivariate statistical correlations between types of maltreatment and bivariate correlations with clinical and sociodemographic variables are presented in Table 3. Most types of maltreatment are significantly mutually correlated, except for sexual abuse with emotional and physical neglect. The data indicate that the highest positive correlations are between emotional abuse and emotional neglect (Spearman’s *ρ* = 0.489, p < 0.01), emotional abuse and physical abuse (Spearman’s *ρ* = 0.377, p < 0.01), and emotional neglect and physical neglect (Spearman’s *ρ* = 0.322, p < 0.01). These correlations show that such types of maltreatment varied together and in the same direction. On the other hand, these correlations are moderate, as Spearman’s rho is distant from the absolute value (*ρ* = 1). Therefore, while there is a statistically significant correlation, the strength of the association between these types of maltreatment is moderate.

**Table 3:** Bivariate Spearman’s rho (*ρ*) correlations between types of maltreatment

Types of maltreatment	Mean (SD)	1. Emotional abuse	2. Physical abuse	3. Sexual abuse	4. Emotional neglect	5. Physical neglect
1. Emotional abuse	9.00 (4.22)	-	0.377*	0.167*	0.489^†^	0.227*
2. Physical abuse	6.25 (2.01)	0.377*	-	0.077^†^	0.264*	0.168*
3. Sexual abuse	5.25 (1.04)	0.167*	0.077^†^	-	0.067	0.075
4. Emotional neglect	9.18 (4.05)	0.489*	0.264*	0.067	-	0.322^†^
5. Physical neglect	5.90 (1.60)	0.227*	0.186*	0.075	0.322*	-

SD = standard deviation.

* Correlation is significant to the 0.05 level (2-tailed)

^†^ Correlation is significant to the 0.01 level (2-tailed)

Table 4 shows the bivariate Spearman’s coefficients for correlations between types of maltreatment and clinical variables. There were no significant correlations between most of the colors/races and the types of maltreatment. The only correlation, though weak, was between those who declared themselves East Asian and sexual abuse (Spearman’s *ρ* = 0.13, p < 0.05). Increased age was subtly correlated with an increase in exposure to emotional neglect (Spearman’s *ρ* = 0.08, p < 0.05), while female sex was correlated with higher levels of emotional abuse (Spearman’s *ρ* = 0.21, p < 0.0005), sexual abuse (Spearman’s *ρ* = 0.08, p < 0.05), and emotional neglect (Spearman’s *ρ* = 0.07, p < 0.05).

**Table 4:** Descriptive variables and Spearman’s coefficients for correlations with the types of maltreatment

Variables	Prevalence (%) or mean (SD) of scores	Types of maltreatment				
		Emotional abuse	Physical abuse	Sexual abuse	Emotional neglect	Physical neglect
Color or race*						
White	14.5%	-0.01	-0.08^†^	0.003	0.003	-0.01
Black	29.7%	-0.02	0.06	0.01	-0.01	-0.06
Multiracial	44.3%	0.04	0.02	-0.07	0.04	0.03
East Asian	6.6%	-0.01	0.07	0.13^‡^	-0.02	0.02
Indigenous	4.6%	-0.007	-0.04	0.02	-0.04	0.02
Sex (female)*	52.2%	0.224^‡^	0.028	-0.095^†^	-0.068	0.050
Age	14.3 (1.8)	0.104^†^	0.115^‡^	0.011	0.066	-0.044
Internalizing symptoms	48.8 (22.0)	0.509^‡^	0.234^‡^	0.153^‡^	0.223^§^	0.129^‡^
Depressive symptoms	9.0 (5.4)	0.54^9^‡	0.268^‡^	0.153^‡^	0.329^‡^	0.188^‡^
Anxiety symptoms	39.8 (17.9)	0.460^‡^	0.205^‡^	0.142^‡^	0.173^‡^	0.097^†^
Intentional self-harm^*^	16.1%	0.29^2^‡	0.191^‡^	0.080^†^	0.201^§^	0.107^‡^
Was bullied^||^	32.3%	0.268^‡^	0.161^‡^	0.085^†^	0.163^‡^	0.099^†^
Bullied others^*¶^	24.3%	0.118^‡^	0.112^‡^	0.061	0.114^‡^	-0.020

SD = standard deviation.

* Transformed into dichotomous variables: color/race (white = 1; non-white = 0, and so on); sex (0 = female; 1 = male); self-harm (yes = 1; no = 0); was bullied and bullied others (yes = 1; no = 0).

^†^ p < 0.05; ^‡^ p < 0.01; ^§^ p < 0.0005.

^||^ Was bullied at least once a week.

^¶^ Bullied others at least once a week.

**Figure 1:**
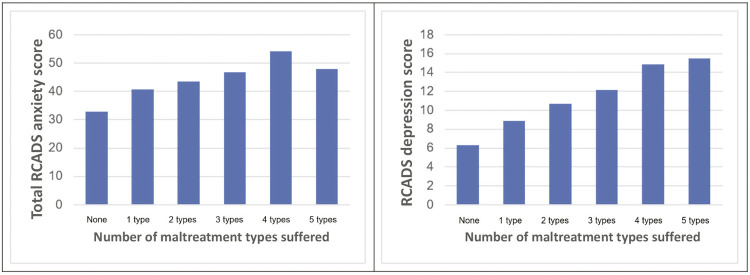
Intensity of anxiety and depression symptoms according to Revised Child Anxiety and Depression Scale (RCADS-47) per number of maltreatment types suffered.

All types of maltreatment were statistically correlated with depressive and anxiety symptoms (internalizing symptoms). The highest correlations were found between depressive symptoms and emotional abuse (Spearman’s *ρ* = 0.549, p < 0.01), between internalizing symptoms and emotional abuse (Spearman’s *ρ* = 0.509, p < 0.01), and between anxiety symptoms and emotional abuse (Spearman’s *ρ* = 0.460, p < 0.01). Figure 1 illustrates correlations between number of types of maltreatment, showing the cumulative effect of abuse and neglect on the intensity of depression and anxiety symptoms. Total scores above 60 for anxiety and above 58 for depression indicate clinical symptoms. Accumulated maltreatments increased these symptoms, but not to the point of reaching the clinical threshold, according to the scores.

Intentional self-harm was correlated with all types of maltreatment, particularly emotional abuse (Spearman’s *ρ* = 0.292, p < 0.01). Being bullied was positively correlated with emotional abuse (Spearman’s *ρ* = 0.268, p < 0.01), and bullying others was correlated with emotional abuse (Spearman’s *ρ* = 0.118, p < 0.01). Moreover, emotional neglect and physical abuse were found to be strongly correlated with psychological and sociodemographic variables, which are detailed in Table 4.

The results of the multiple linear regression analyses are shown in Tables 5 and 6. Each type of maltreatment was analyzed alone (i.e., without the inclusion of covariables) as a predictor of anxiety and depressive symptoms in RCADS-47. Then, all types of maltreatment were simultaneously considered as predictors of the symptoms to identify the strongest predictors. Both analyses included sex and age as covariables.

**Table 5:** Associations between types of maltreatment and anxiety and depressive symptoms

Psychological symptoms	Regression models							

	Only one type of maltreatment as predictor				All types of maltreatment as predictors			

	β	Standardized β	95%CI		β	Standardized β	95%CI	
	
			LL	UL			LL	UL
Social anxiety								
Emotional abuse	0.37*	0.28	0.28	0.47	0.338^†^	0.25	0.21	0.45
Physical abuse	0.57^†^	0.20	0.36	0.77	0.301*	0.10	0.07	0.52
Sexual abuse	0.51^‡^	0.09	0.11	0.91	0.23	0.04	-0.16	0.62
Emotional neglect	0.16*	0.12	0.06	0.27	-0.03	-0.02	-0.15	0.08
Physical neglect	0.22*	0.06	-0.04	0.48	-0.06	-0.01	-0.34	0.20
Panic								
Emotional abuse	0.46^†^	0.38	0.38	0.54	0.45^†^	0.38	0.35	0.55
Physical abuse	0.39^†^	0.15	0.21	0.57	-0.02	-0.008	-0.20	0.16
Sexual abuse	0.64^†^	0.13	0.29	0.98	0.39^‡^	0.08	0.07	0.72
Emotional neglect	0.25^†^	0.18	0.13	0.31	-0.02	-0.01	-0.12	0.08
Physical neglect	0.37*	0.11	0.14	0.60	0.05	0.01	-0.16	0.28
Separation anxiety								
Emotional abuse	0.16^†^	0.21	0.10	0.21	0.15^†^	0.20	0.08	0.21
Physical abuse	0.17*	0.11	0.06	0.28	0.02	0.01	-0.09	0.14
Sexual abuse	0.34*	0.11	0.12	0.56	0.24^‡^	0.08	0.02	0.46
Emotional neglect	0.06^‡^	0.08	0.006	0.12	-0.03	-0.04	-0.10	0.03
Physical neglect	0.20*	0.10	0.06	0.34	0.11	0.05	-0.03	0.26
Generalized anxiety								
Emotional abuse	0.32^†^	0.33	0.25	0.39	0.34^†^	0.35	0.25	0.43
Physical abuse	0.33^†^	0.16	0.18	0.48	0.06	0.03	-0.09	0.22
Sexual abuse	0.51*	0.13	0.22	0.80	0.29^‡^	0.07	0.01	0.57
Emotional neglect	0.10*	0.10	0.02	0.17	-0.08^‡^	-0.08	-0.17	-0.003
Physical neglect	0.22^‡^	0.08	0.02	0.41	0.02^‡^	0.01	-0.16	0.22
Obsessive-compulsive								
Emotional abuse	0.33^†^	0.36	0.27	0.40	0.34^†^	0.36	0.26	0.43
Physical abuse	0.29^†^	0.15	0.14	0.44	0.002	0.001	-0.15	0.15
Sexual abuse	0.50^†^	0.13	0.22	0.79	0.31^‡^	0.08	0.04	0.58
Emotional neglect	0.13^†^	0.14	0.06	0.21	-0.05	-0.05	-0.13	0.03
Physical neglect	0.26*	0.10	0.07	0.45	0.05	0.02	-0.13	0.24

95%CI = 95% confidence interval; LL = lower limit; UL = upper limit.

* p < 0.001; ^†^ p < 0.0005; ^‡^ p < 0.05.

**Table 6:** Associations between types of maltreatment and anxiety and depressive symptoms

Psychological symptoms	Regression models							

	Only one type of maltreatment as predictor				All types of maltreatment as predictors			

	β	Standardized β	95%CI		β	Standardized β	95% CI	
	
			LL	UL			LL	UL
Depression								
Emotional abuse	0.67*	0.52	0.59	0.75	0.56*	0.43	0.46	0.66
Physical abuse	0.74*	0.27	0.55	0.94	0.185	0.06	-0.001	0.37
Sexual abuse	0.71*	0.13	0.33	1.09	0.334^†^	0.167	0.006	0.66
Emotional neglect	0.43*	0.32	0.34	0.52	0.09	0.05	-0.005	0.19
Physical neglect	0.69*	0.20	0.44	0.93	0.151	0.04	-0.07	0.37
Total anxiety								
Emotional abuse	10.66*	0.39	1.37	1.95	1.63*	0.38	1.28	1.99
Physical abuse	0.76*	0.19	1.12	2.41	0.37	0.04	-0.28	1.03
Sexual abuse	20.51*	0.14	1.27	3.76	1.44^†^	0.08	0.027	2.62
Emotional neglect	0.70*	0.15	0.38	1.02	-0.22	-0.05	-0.58	0.12
Physical neglect	10.29^‡^	0.11	0.46	2.11	0.16	0.01	-0.64	0.98
Internalizing symptoms								
Emotional abuse	20.33*	0.44	1.99	2.68	2.19*	0.42	1.77	2.62
Physical abuse	20.51*	0.22	1.73	3.28	0.55	0.05	-0.22	1.37
Sexual abuse	30.23*	0.15	1.72	4.74	1.82^†^	0.08	0.44	3.20
Emotional neglect	10.13*	0.20	0.74	1.52	-0.13	0.215	-0.56	0.28
Physical neglect	1.98*	0.14	0.98	2.97	0.33	0.02	-0.61	1.29

95%CI = 95% confidence interval; LL = lower limit; UL = upper limit.

* p < 0.0005; ^†^ p < 0.05; ^‡^ p < 0.001.

In the models with all types of maltreatment as covariables, emotional abuse was the strongest predictor of anxiety and depressive symptoms: depression (β = 0.56, p < 0.0005); total anxiety (β = 1.63, p < 0.0005). Specifically with regard to the anxiety symptoms, a 1-unit increase on the emotional abuse scale (CTQ) predicts a 1.63-point increase in the total RCADS-47 anxiety score. Sexual abuse also emerged as a predictor, although weaker, of total anxiety (β = 1.44, p < 0.05) and depressive symptoms (β = 0.334, p < 0.05).

Table 7 presents the results of the binary logistic regression analysis, which assessed the predictive power of maltreatment for drug use, bullying others, and engaging in self-harming behavior.

**Table 7:** Binary logistic regression for prediction of the probability of risk behaviors based on maltreatment

Risk behaviors	B	OR	OR (95%CI)	

			Lower limit	Upper limit
Alcohol use				
Sex (male)	0.11	1.12	0.79	1.57
Age	0.43*	1.53	1.39	1.7
Emotional abuse	0.05^†^	1.05	1.00	1.11
Physical abuse	0.11^†^	1.12	1.02	1.24
Sexual abuse	0.10	1.10	0.92	1.32
Emotional neglect	0.007	1.00	0.95	1.05
Physical neglect	-0.08	0.91	0.81	1.03
Cannabis use				
Sex (male)	0.58	1.79	0.7	4.5
Age	0.13	1.14	0.82	1.59
Emotional abuse	0.03	1.04	0.88	1.22
Physical abuse	-0.20	0.81	0.53	1.24
Sexual abuse	0.22	1.24	0.82	1.89
Emotional neglect	0.09	1.09	0.93	1.29
Physical neglect	-0.08	0.92	0.60	1.41
Other substance use				
Sex (female)	0.53	0.58	0.05	5.9
Age	-0.06	0.93	0.54	1.6
Emotional abuse	0.18	1.20	0.94	1.52
Physical abuse	-0.12	0.88	0.49	1.59
Sexual abuse	0.09	1.10	0.57	2.1
Emotional neglect	0.03	1.03	0.77	1.38
Physical neglect	-0.28	0.75	0.33	1.67
Intentional self-harm				
Sex (female)	0.71^‡^	0.49	0.3	0.8
Age	-0.12^†^	0.88	0.77	0.99
Emotional abuse	0.12*	1.13	1.06	1.2
Physical abuse	0.10	1.1	0.99	1.23
Sexual abuse	0.08	1.09	0.91	1.3
Emotional neglect	0.05	1.05	0.99	1.12
Physical neglect	-0.05	0.95	0.82	1.09
Bullying others				
Sex (male)	0.80*	2.24	1.53	3.27
Age	0.05	1.05	0.95	1.16
Emotional abuse	0.07*	1.07	1.02	1.13
Physical abuse	0.008	1	0.91	1.1
Sexual abuse	-0.00	0.99	0.83	1.19
Emotional neglect	0.02	1.02	0.97	1.08
Physical neglect	-0.10	0.9	0.79	1.02

95%CI = 95% confidence interval; LL = lower limit; OR = odds ratio; UL = upper limit.

* p < 0.0005; ^†^ p < 0.05; ^‡^ p < 0.01.

The binominal logistic regression indicated that certain variables have predictive power for adolescent alcohol use, self-cutting, and bullying others, particularly for those who have suffered emotional abuse. The risk of adolescents using alcohol increased 1.53 times with an increase in age, and 1.05 to 1.12 times if they had suffered emotional or physical abuse, respectively.

The risk of adolescents cutting themselves intentionally increased 1.13 times if they had suffered emotional abuse, 0.88 times if they were younger, and 0.49 times if they were female. The risk of bullying others increased 2.24 times if they were male adolescents, and 1.07 times if they had suffered emotional abuse.

## Discussion

This study showed that emotional abuse and emotional neglect are the most frequent types of maltreatment in the population studied, with 42.4 and 39% prevalence respectively. This study replicated the methods used by Cecil et al.^6^ and Oliveira et al.^24^ The study by Cecil et al.^6^ assessed 16-to-24-year-old people in social vulnerability. The most prevalent maltreatments were emotional neglect (49.5%) and emotional abuse (48%).

A previous study by our group^24^ analyzed a sample with a similar age range (11 to 18 years old) but more socially vulnerable, given that the school was located in a violent neighborhood. The most common maltreatments were emotional neglect (45.2%) and physical neglect (41.7%). Data from both prior studies are similar to those from the present study. The sample in the present study showed a lower frequency of maltreatment related to physical neglect, which may be due to this population being less exposed to poverty and violence. On the other hand, emotional abuse was high in all three studies.

These data are interesting in that they agree with other studies^25,35^ that indicate a high frequency of emotional abuse and neglect.^36^ Therefore, these types of maltreatment should be addressed with prevention and intervention strategies targeting affected populations. The high frequency of abuse and neglect may be explained by offensive language and criticism and abusive caregivers who maintain harmful patterns, unaware of their deleterious effects. Furthermore, such deleterious effects are not immediately perceivable, as in the case of physical abuse and neglect.^36^ Some cultures and societies may even facilitate emotional abuse in minorities, such as young people who are exposed to criticism due to their physical vulnerability.^37^

Confirming the damage caused by these types of maltreatment, our data agree with the current literature regarding the deleterious effects of these types of childhood maltreatment.^38,39^ Emotional abuse was the variable most correlated with and predictive of anxiety and depressive symptoms and risk behaviors, such as self-cutting and psychoactive substance use.^40^

Our findings also showed that emotional abuse co-occurs with other types of maltreatment. Hence, it can be considered a nonspecific marker and should therefore be emphasized when studying other forms of abuse, such as sexual and physical abuse.^41^ On the other hand, other studies^42,43^ highlight the specific role of emotional abuse in the development of psychological changes – which reinforces the data described here. Moreover, various studies in different cultures and populations^44,45^ point out that this category makes people vulnerable to development of mental disorders.

Our data indicate that the higher the level of emotional abuse, the more severe the anxiety and depressive symptoms and behaviors, such as bullying others and self-harming – which had the strongest correlations in the data. Besides emotional abuse, there is evidence that emotional neglect and physical abuse influence the occurrence of depressive symptoms. Other studies^46,47^ have identified associations between these three types of abuse and depression and anxiety disorders in adulthood.

Bullying others occurred together with emotional abuse and emotional neglect, which indicates that people (especially men) who perpetrate bullying may need more psychological attention. Data in this study indicate that having suffered emotional abuse increased by more than one time the chance of men perpetrating bullying. Likewise, it has been observed that people who committed violence had also suffered all types of maltreatment.^24,48^

The maltreatment types and social variables were included as covariables in the analysis to assess which was the preponderant factor in predicting the variables of interest. We found that emotional abuse and sexual abuse were the greatest predictors of RCADS-47 total anxiety and depression scores. The data also indicated that female gender was positively correlated with emotional abuse, sexual abuse, and neglect. Data from other studies emphasize the occurrence of such abuse in women, particularly in Brazil and other Latin American countries.^36,49^ Additionally, our study indicates that being female increases the risk of self-mutilation. In this sample, in which age ranged from 11 to 17 years, the youngest participants were more likely to harm themselves. These data are compatible with other research indicating that females are more prone to self-harm, as well as showing the early age at which these risk behaviors begin.^38,50^

Some of the limitations described in our previous article (de Oliveira et al.^24^) are also observed in the present study. First, the main instrument used to measure maltreatment (CTQ) was a self-report measure, which is known to be susceptible to retrospective memory bias. Also, self-report measures do not inform about experiences of child maltreatment that occurred at early stages of development. However, the findings of the present study are consistent with both the UK study (Cecil et al.^6^) and our prior study replicating it (de Oliveira et al.^24^), which makes the associations observed more reliable. A second limitation was that the measure we used to assess maltreatment prevented us from detecting which specific aspects of emotional abuse produced psychiatric symptomatology, suggesting more specific measures of maltreatment should be used in the future. A third limitation was that the results are based on cross-sectional and associational data. Finally, because parents were also invited to participate to provide information, it is possible that the participants were reluctant to disclose drug use, which might explain the low rate of reported drug use.

In conclusion, emotional abuse was the most prevalent, the most strongly correlated, and the greatest predictor of anxiety, depression, and risk behaviors. Moreover, emotional neglect and sexual abuse stand out as noteworthy risk factors that make subjects vulnerable. This study indicated that adolescents are more exposed to maltreatment, especially emotional abuse. This emphasizes the need for public health promotion and care policies aimed at preventive intervention programs at both home and school. Our data also confirmed that different forms of abuse tend to co-occur and correlate with each other, which increases the probability of developing anxiety and depressive symptoms, regardless of the type.
